# The General Requirements of a Scientific Medical Manuscript

**DOI:** 10.34172/aim.2022.79

**Published:** 2022-08-01

**Authors:** Mohammad Hossein Azizi, Shokoofeh Borzabadi

**Affiliations:** ^1^Associate Editor, Academy of Medical Sciences IR, Iran, Tehran, Iran; ^2^Executive Director, Copy Editor, Academy of Medical Sciences IR, Iran, Tehran, Iran

 The first issue of the *Archives of Iranian Medicine* (AIM) was published in 1998. AIM is a multi-disciplinary general medical English publication which mostly covers clinical subjects. It is an open-access, free-of-charge journal. In 2021, we received 2209 papers and only 167 (7.6%) were accepted. All received papers are peer-reviewed by experts.

 In order to remind some important issues to our respected readers and researchers who intend to submit their papers, the following points are highlighted. However, for more information, one must best refer to the instructions for authors (http://www.aimjournal.ir/InstructionsforAuthors).

- All submitted papers are initially screened for technical flaws and some of them may be desk-rejected due to various reasons including lake of novelty, inappropriate format, poorly written manuscript, incomplete structure of the paper and possible plagiarism. It seems that some authors ignore the aim and scope of our journal!
- The first part of a paper which is initially noticed by the editorial team and reviewers is the title. Accordingly, it should be brief, clear and reasonable, preferably usually not more than twenty words. It is best to avoid using abbreviations in the title. If necessary, only known and standard abbreviations could be used. The title should not be misleading
- The second important part of an article which demonstrates the gist of the research is the abstract. The editorial team and reviewers consider it as a fundamental part of a paper. The main message of the paper should be clearly mentioned. Therefore, the abstract is comprehensive and contain the important details of the research in no more than 250 words. For original articles, based on our journal’s format, the abstract is structured and the sample size (number of patients or subjects) also included. The keywords should be selected only based on MeSH.
- In the Introduction, the main message of the study should be included.
- The results of the research should be consistent with its objective.
- Ethical considerations, including informed consent from patients, are necessary when applicable.
- The methodology of the original articles and systematic reviews should be scientifically valid and accurate; otherwise the biostatistician reviewers may reject them.
- The Discussion should be conclusive and logical.
- All submitted papers will be peer-reviewed by internal or external peer-reviewers and this process takes time (sometimes up to 3 months). If possible, introduce three competent and unbiased peer-reviewers. It should be mentioned that only the novel up-to-date papers which are written by distinguished experts are placed on the fast tract.
- In order to facilitate the process of paper acceptance, the corresponding authors should answer the comments of the reviewers properly as soon as possible (maximum two weeks) in the form of a precise covering letter. Otherwise it may result in delays in publication or even final rejection.
- References should be up-to-date when possible.
- We are a member of the COPE (Committee on Publication Ethics) and accordingly, the papers which are submitted to the AIM journal should be consistent with the standards of the COPE. Fabricated or plagiarized papers will be strongly rejected. All papers are initially screened for possible plagiarism. Avoid high proportions of self-citation from your previous works.
- Before submission, check your article for typos, punctuation and grammar errors, because they are not acceptable in a valid scientific paper.
- Resubmission of a previously rejected paper is not acceptable.
- We highly suggest that do not submit the paper to another journal until the end of the evaluation process.

Rejection of some papers does not mean that they are worthless, but it may mean that the journal authorities could not adopt it to the journal’s priorities due to the reasons stated above.

 Last but not least, the editorial team and reviewers are always ready to help the authors in order to improve the manuscripts scientifically.
